# Evaluating CK20 and MCPyV Antibody Clones in Diagnosing Merkel Cell Carcinoma

**DOI:** 10.1007/s12022-024-09845-w

**Published:** 2025-01-22

**Authors:** Begum Yeni Erdem, Can Baykal, Yasemin Ozluk, Melin A. Ahmed, Erol Kozanoglu, Pinar Saip, Nesimi Buyukbabani, Sule Ozturk Sari

**Affiliations:** 1 Department of Pathology, Istanbul Faculty of Medicine, Istanbul University, Millet Caddesi, Fatih, Istanbul, 34093 Turkey; 2 Department of Dermatology and Venereology, Istanbul Faculty of Medicine, Istanbul University, Millet Caddesi, Fatih, Istanbul, 34093 Turkey; 3 Department of Medical Oncology, Institute of Oncology, Istanbul University, Millet Caddesi, Fatih, Istanbul, 34093 Turkey; 4 Department of Plastic, Reconstructive and Aesthetic Surgery, Istanbul Faculty of Medicine, Istanbul University, Millet Caddesi, Fatih, Istanbul, 34093 Turkey; 5 Present Address: Department of Pathology, Koc University Hospital, Davutpasa Caddesi, Zeytinburnu, Istanbul, 34010 Turkey

**Keywords:** Merkel cell carcinoma, Cytokeratin 20, Merkel cell polyomavirus, Clone Ab3, Clone CM2B4

## Abstract

Merkel cell carcinoma (MCC) is diagnosed through histopathological and immunohistochemical examination of biopsies from skin or other organs. Its distinguishing features include perinuclear dot-like staining with Cytokeratin 20 (CK20) and detection of Merkel cell polyomavirus (MCPyV) using various methods. However, CK20 and MCPyV negative MCC cases have been reported at varying rates. In this single center cross-sectional study, we aimed to determine which clones are more effective in diagnosing MCC by comparing the performance of CK20 antibody clones Ks20.8 and SP33, as well as MCPyV antibody clones Ab3 and CM2B4. Fifty-four patients diagnosed with MCC were included. Among these, 42 cases were primary cutaneous, and 12 cases were nodal MCC. Fifty-two (96.3%) cases were positive with both CK20 clones, while two cases were negative. Clone SP33 stained areas of necrosis, whereas Ks20.8 showed no aberrant staining. MCPyV was detected in 44 cases (81.5%) using clone Ab3 and 39 cases (72.2%) using clone CM2B4. Staining with MCPyV clone Ab3 was diffuse and strong in most cases, while approximately 30% of CM2B4-positive cases exhibited low percentages and/or weak staining, complicating the evaluation. The two CK20-negative cases were also negative with both MCPyV clones. Our data demonstrated that CK20 clone Ks20.8 may be preferred for MCC diagnosis due to its consistent performance and lack of aberrant staining. Similarly, MCPyV clone Ab3 appears superior to CM2B4 for identifying MCPyV-positive cases.

## Introduction

Merkel cell carcinoma (MCC) is a rare and aggressive primary neuroendocrine carcinoma of the skin [1]. The highest number of patients is reported from North America, while Australia has the highest incidence in the world with a rate of 1.6 per 100,000 population [2, 3]. The incidence rate in Europe is much lower at 0.13 per 100,000 [4]. However, a common trend seen in all of these regions is that the incidence of MCC increases with societal ageing and the prevalence of immunosuppressive drug use [2–5].

Merkel cell polyomavirus (MCPyV) and sun damage are the main factors playing role in the etiology of MCC, and the contribution of these factors varies according to geographical regions [1, 6–8]. In Australia and New Zealand, sun damage predominantly contributes to the etiology, while in other parts of the world, MCPyV is known to be more influential [9–11]. Skin lesions are most commonly localized in the head and neck region and extremities [12, 13]. But patients may rarely present with lymph node metastasis without a primary skin tumor [14–16]. Since the histopathology of MCC is similar to that of small round cell tumors, immunohistochemical stains including neuroendocrine markers should be used for proper identification. Unlike other neuroendocrine tumors, perinuclear dot-like staining with Cytokeratin 20 (CK20) is MCC’s distinguishing diagnostic hallmark [17, 18]. In addition to the dot-like staining, crescent-shaped and, less frequently, membranous staining may also be observed [19, 20]. In nearly all reported series until now the clone Ks20.8 was used and CK20 negativity was rarely encountered (approximately 10%) which may cause difficulty in the exclusion of metastatic neuroendocrine carcinoma originating from other organs [17, 18]. Especially in these cases, clinical and radiological investigation of other potential origins of neuroendocrine tumors is crucial.

Another helpful diagnostic tool is the detection of MCPyV, which is a specific feature of MCC [6, 19]. Using various viral pathogen identification techniques, most commonly quantitative real-time PCR, MCPyV has been shown to be positive in approximately 24–85% of MCC cases [9, 21–24]. The virus was first demonstrated immunohistochemically in 2009, using the MCPyV antibody clone CM2B4 [25]. Subsequent studies comparing the immunohistochemical method with PCR demonstrated a good correlation between the two methods [19, 26]. In 2012, a new clone of the MCPyV antibody, termed Ab3, was found to show higher sensitivity than CM2B4 [27]. Both clones were developed based on the peptide sequence of exon 2 in the LTag of the virus, which is unique for MCPyV [28]. CM2B4 binds to amino acids 116–129, while Ab3 targets the region spanning amino acids 79–260 [21, 25, 27].

The advantages and disadvantages of these two commercially available clones have only been investigated in a limited number of studies, and a gold standard method for MCPyV detection has yet to be established [21, 29, 30]. Besides, no comparative study of CK20 antibody clones has been conducted. In our study, we used clone SP33 alongside clone Ks20.8 to assess whether the infrequently observed CK20 negativity was related to the antibody clone used. Additionally, we examined the advantages and disadvantages of two commercially available MCPyV antibody clones, namely Ab3 and CM2B4.

## Materials and Methods

### Case Selection

The single center cross-sectional study received institutional review board approval (Istanbul Tip Fakultesi Klinik Arastirmalari Etik Kurulu, number 256889, date 24.06.2021). Between 2002 and 2022, 67 biopsy and/or excision materials diagnosed as MCC were identified in our archive. Among these, 54 cases with available paraffin blocks and proper fixation allowing morphologic and immunohistochemical evaluation were included in the study.

In 42 cases, the primary tumor was located in the skin, while in 12 cases, it was located in a lymph node. In these 12 cases, no significant skin lesion was found upon detailed physical examination and no other neuroendocrine carcinoma was detected on radiological investigations. The diagnosis was further supported with the previously described “ELECTHIP criteria” of MCC of lymph nodes-nodal MCC [15].

### Histopathological Evaluation

Hematoxylin–eosin (HE) stained slides of the cases were re-examined. Cell shape, cytoplasmic features, nucleolar prominence and chromatin structure, structural features of the tumor and distinct areas of differentiation, if any, were evaluated and noted by two pathologists (BYE, SOS).

### Immunohistochemical Method

In all cases, epithelial and neuroendocrine markers were routinely used in the diagnostic workup. For the study, a paraffin block with the most representative tumor and optimum fixation was determined for each case. Immunohistochemistry was performed using the automated Ventana Medical System-Benchmark XT IHC/ISH Staining System.

Control tissue blocks were created in order to optimize the staining method. Colonic adenocarcinoma tissue was used as positive external control for CK20 [31]. Since CK20 clone Ks20.8 had been routinely applied to all cases in the initial diagnostic workup, only negative cases were repeatedly stained. There were no controls other than MCC for MCPyV antibodies. Optimization trials were performed on several blocks of three study cases. Ideal dilution and incubation times were determined. Table 1 shows the characteristics of the markers.

**Table 1:** Technical details of primary antibodies used for immunohistochemical analysis

Antibody	Manufacturer	Clone	Dilution	Incubation time
CK20 – Mouse monoclonal	Cell Marque	Ks20.8	1:100	32 min
CK20 – Rabbit monoclonal	Ventana	SP33	Ready to use	32 min
MCPyV – Mouse monoclonal	Abcam	Ab3	1:200	60 min
MCPyV – Mouse monoclonal	Santa Cruz	CM2B4^*^	1:50	120 min

^*^ An amplifier was used

*CK20* Cytokeratin 20; *MCPyV* Merkel cell polyomavirus

### Immunohistochemical Evaluation

#### CK20 (for Both Clones Ks20.8 and SP33)

We looked for perinuclear dot-like (DL), crescent-shaped (CS) and membranous (M) staining pattern. Staining patterns, intensity and extent of staining were separately evaluated in all cases.

#### MCPyV (for Both Clones Ab3 and CM2B4)

Nuclear staining was evaluated [25, 27]. In some cases, cytoplasmic staining was observed alongside nuclear staining. Cytoplasmic staining alone was considered negative. Cases were considered positive according to the criteria of at least weak staining above 1% of cells used in the literature (Allred score > 2) [21, 22].

### Statistical Analysis

Statistical Package for Social Sciences (SPSS) for Mac 24.0 package was used in statistical analyses. Descriptive statistics were presented as frequencies (n) and percentages (%) for categorical variables, while numerical variables were expressed using the mean, minimum, and maximum values.

## Results

### Clinical Findings

Fifty-four patients (26 female, 28 male) were between the ages of 24–91 years, with a mean age of 67 years. Among the patients, five were immunosuppressed, including the 24-year-old patient. Four of these were immunosuppressed due to organ transplantation, and one due to treatment for rheumatoid arthritis. The clinical features of three patients with organ transplantation were previously reported in a multicenter study [32]. In 42 cases of primary cutaneous MCC, the most common site was the extremities (50%), with the remainder distributed in the head and neck (33%) and trunk (17%). The involved lymph nodes of 12 nodal MCC patients were inguinal (83.3%), axillary (one case) and intraparotid lymph nodes (one case).

### Histopathological Findings

**Fig. 1:**
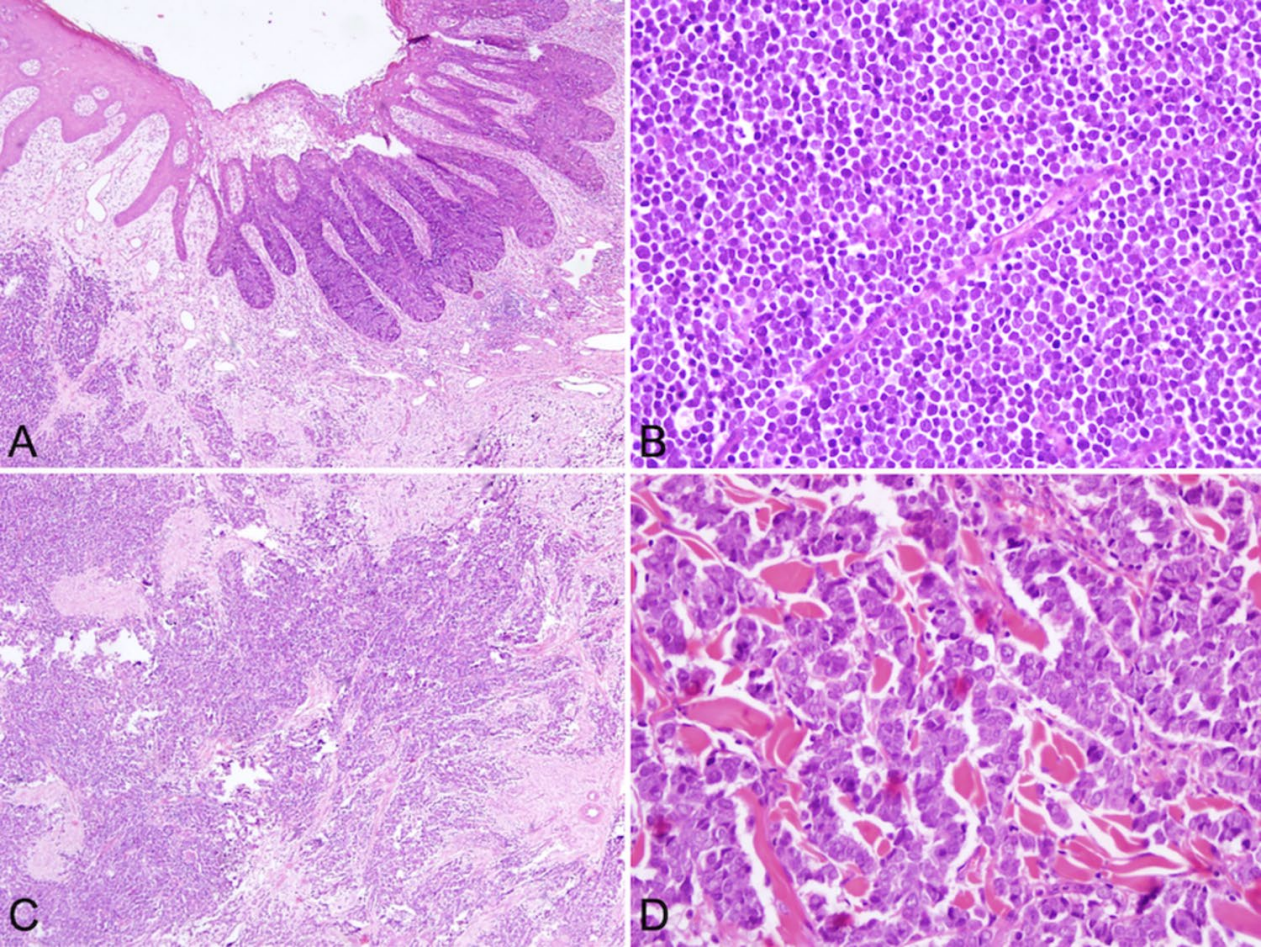
Histopathological findings: **A** and **C** Bowen disease over MCC; **B** Round shaped cells with narrow cytoplasm; **D** Anastomosing and irregular trabecular configuration of tumor cells with open chromatin

Tumor cells were mostly round shaped, with narrow cytoplasm. Salt & pepper chromatin was visible in all cases and nucleoli were indistinct. Solid and trabecular arrangements were present in the tumors at varying rates. Necrosis was observed in approximately 70% (36/54) of cases. All cases were pure MCC, except for one case where MCC was associated with SCC in situ (Bowen disease) (Fig. 1).

### Immunohistochemical Findings

#### CK20

Only two (3.7%) cases were negative with both clones of CK20 (Ks20.8 and SP33). All remaining cases were positive with both clones. MCC associated with Bowen disease was CK20 positive. Among the positive cases, the CS + M + DL pattern was observed in 73.1% (38/52) and 59.6% (31/52) for clones Ks20.8 and SP33, respectively. In 35 cases, M, CS and DL stainings were identical for both clones, and distributed as follows; M + CS + DL in 7 cases, CS + DL in 14 cases and DL only in 14 cases. In the remaining 17 cases, staining patterns differed between the clones. Specifically, while CS + DL staining was observed with Ks20.8 in 7 cases, only DL staining was observed with SP33. In 10 cases M + CS + DL staining was observed with Ks20.8, whereas CS + DL staining was observed with SP33.

In terms of staining intensity, 23 cases were strongly positive with both clones. The staining was stronger with Ks20.8 in 22 cases and with SP33 in three cases. In four cases, the staining was weak with both clones. In reviewing the 17 cases that showed different staining patterns with the clones, we noticed that in 13 cases, the staining was stronger with Ks20.8, and CS + M stainings were added to the DL staining.

**Fig. 2:**
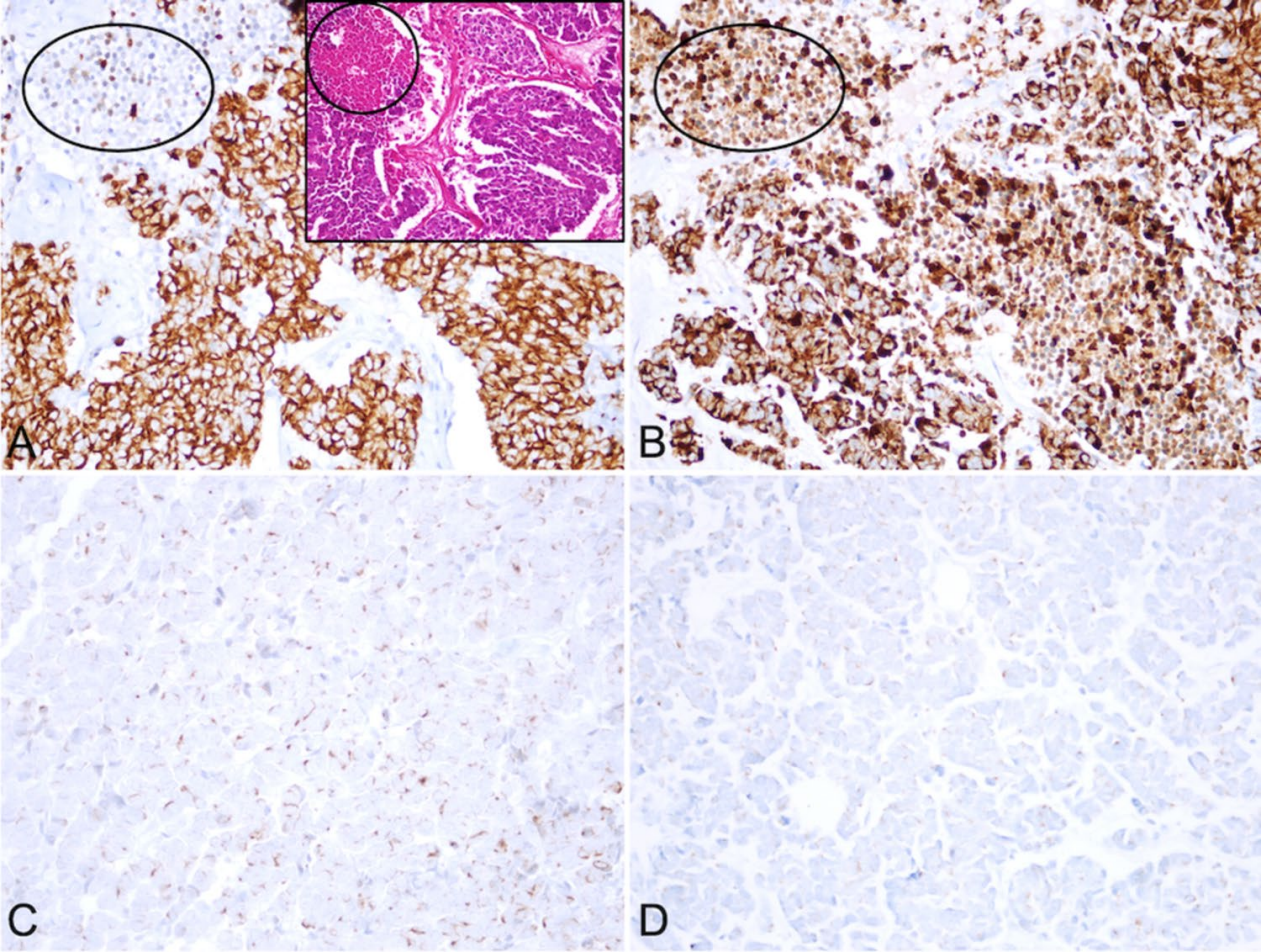
Staining patterns of two clones of CK20 antibody in the same cases: **A-B** Staining in necrosis (**inlet:** H&E; **circles:** areas of necrosis; **A** Ks20.8; **B** SP33); **C-D** Differences in intensity (**C** Ks20.8 stronger; **D** SP33 weak)

Thirty-eight cases showed diffuse staining with both clones of CK20. In 6 cases, the staining with SP33 was patchy, whereas Ks20.8 was diffuse. There were 7 cases showing patchy staining with both clones and one case showed focal staining with both clones. Considering background stainings may complicate the evaluation, we noticed that SP33 stained areas of necrosis in 15 cases (Fig. 2). Within the same areas, there were no aberrant staining with Ks20.8.

#### MCPyV

MCPyV clone Ab3 was positive in 44 cases (81.5%) and MCPyV clone CM2B4 in 39 cases (72.2%). The comparison of cases stained with the two clones is presented in Table 2. There were no cases that stained with CM2B4 but not with Ab3. Most of the Ab3-positive cases showed diffuse and strong staining, while only 44% of CM2B4-positive cases showed diffuse and strong staining. In some of the cases with strong staining, cytoplasmic staining was also present alongside nuclear staining with both clones.

**Table 2:** Comparison of MCPyV clones (CM2B4 vs. Ab3)

**MCPyV**	**Clone Ab3** Diffuse and strong staining	≥ 50% weak staining	< 50% weak staining	Negative	Total number of cases
**Clone CM2B4** Diffuse^*^ and strong staining	17	0	0	0	17
≥ 50% weak staining	10	0	0	0	10
< 50% weak staining	11	1	0	0	12
Negative	3	1	1	10	15
Total number of cases	41	2	1	10	54

^*^ Diffuse staining represents positivity in almost 100% of tumor cells

*MCPyV* Merkel cell polyomavirus

**Fig. 3:**
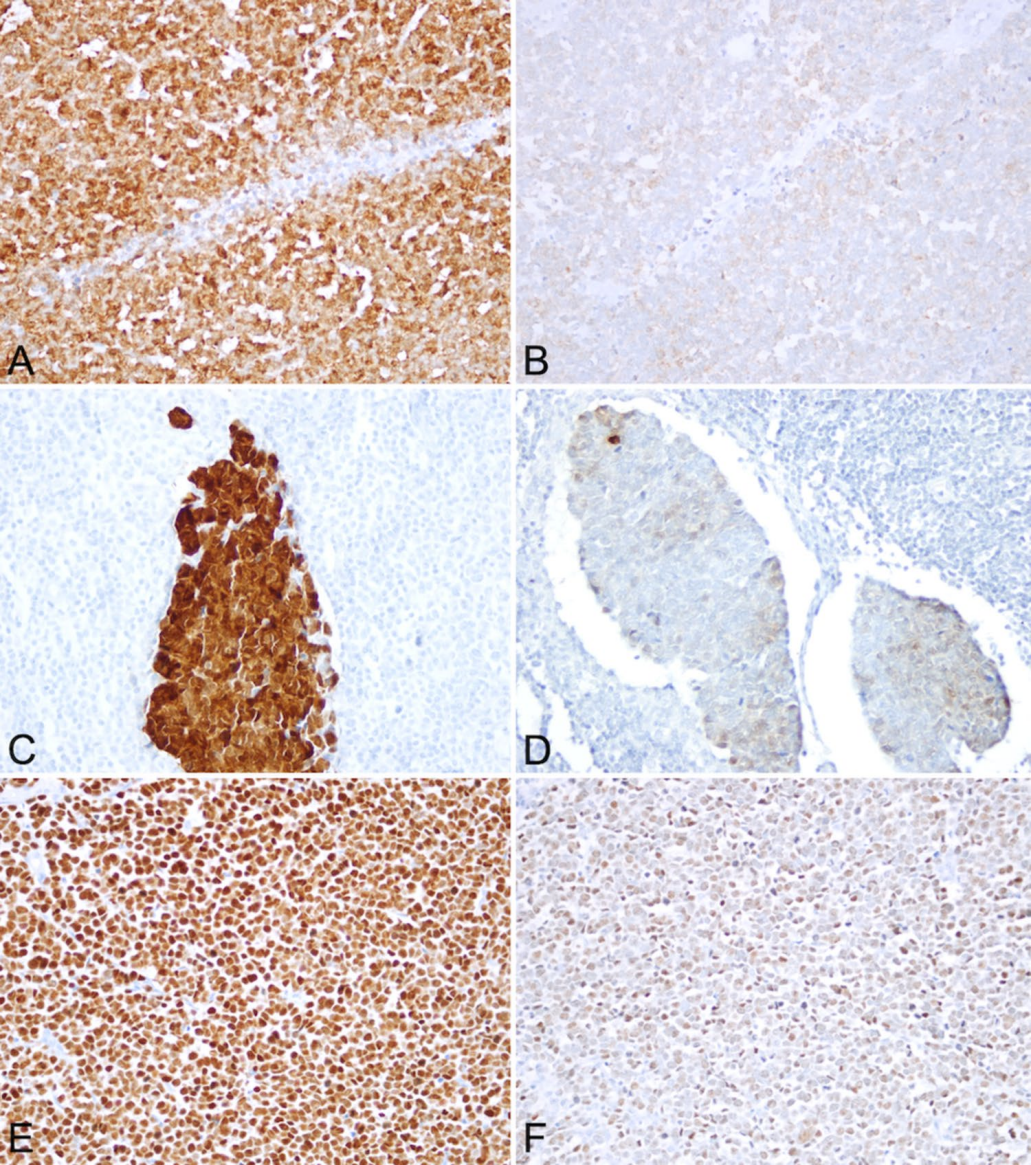
Staining patterns of two clones of MCPyV antibody in the same cases: **A** Ab3 (+); **B** CM2B4 (-) (Cytoplasmic reaction); **C** Ab3 (+); **D** CM2B4 (+) but focal;** E** Ab3 (+); **F** CM2B4 (+) but weak

Ab3 did not show cytoplasmic staining alone in any of the cases, whereas CM2B4 showed cytoplasmic staining alone in one case. This case, which was considered negative with CM2B4, displayed nuclear staining with Ab3 (Fig. 3). No aberrant staining was found in any non-tumoral cell for both clones of MCPyV.

**Fig. 4:**
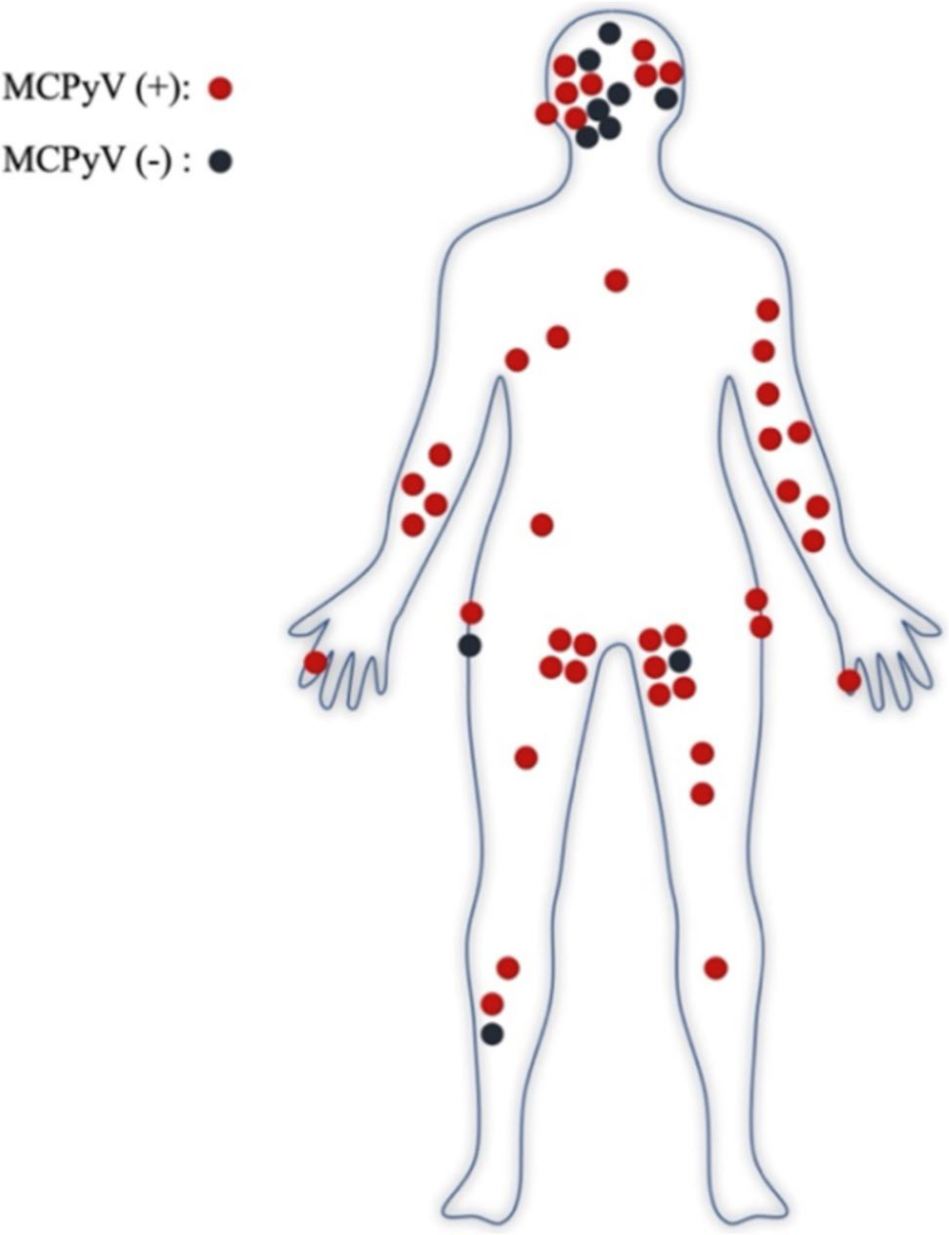
Distribution of primary tumors on a body figure according to MCPyV clone Ab3

Table 3 summarizes the clinicopathological characteristics of the study cohort based on MCPyV positivity with two different antibody clones. Tumor locations on a body figure according to MCPyV clone Ab3 are shown in Fig. 4.

**Table 3:** Clinicopathological characteristics of the study cohort based on MCPyV positivity with two different antibody clones

	MCPyV Ab3 positive cases, *n* (%)	MCPyV CM2B4 positive cases, *n* (%)
**Patient** **immune** **status** (*n*)		
Immunocompetent (49)	40 (82)	35 (71)
Immunosuppressed (5)	4 (80)	4 (80)
**Tumor** **locations** (*n*)		
Cutaneous (42)	33 (79)	28 (67)
Nodal (12)	11 (92)	11 (92)
**Histological** **subtypes** (*n*)		
Pure (53)	44 (83)	39 (74)
Combined (1)	0	0
**CK20** **status** (*n*)		
CK20 (+) (52)	44 (85)	39 (75)
CK20 (-) (2)	0	0

*CK20* Cytokeratin 20; *MCPyV* Merkel cell polyomavirus

## Discussion

In this study, reviewing 54 MCC cases, MCPyV and CK20 antibody clones used in the diagnosis were evaluated. MCPyV clone Ab3, although relatively understudied in the literature, demonstrated a higher positivity rate and was easier to evaluate compared to CM2B4. CK20 clone SP33, which has not been previously reported in MCC series in the literature, demonstrated aberrant staining in areas of necrosis. We observed no difference between clones SP33 and Ks20.8 in their ability to detect positive cases.

MCC, a rare and aggressive neuroendocrine skin carcinoma, has an etiology linked to MCPyV and sun damage, with notable geographical variations [33]. In our series, MCPyV positivity was observed in nearly 80% of cases, a rate comparable to those reported in Europe, where MCPyV is accounted as the major etiologic agent and may suggest that MCC in our region is more influenced by MCPyV than sun damage [24, 34, 35].

For MCPyV detection, a gold standard method has not been established. Since its discovery by Feng et al. in 2008, PCR-based techniques have been widely used due to their sensitivity in detecting MCPyV [22, 36]. It has been demonstrated that MCPyV contributes to tumorigenesis only after integrating into the host genome and undergoing specific mutations in MCC [37]. However, MCPyV has also been detected in non-MCC skin tumors and other malignancies where it does not play a role in pathogenesis, raising concerns about the specificity of PCR in these cases [22, 25, 38, 39].

The MCPyV antibody clone CM2B4, introduced in 2009, showed no reactivity in non-MCC tumors according to current studies [22, 25]. The lack of false positivity with CM2B4 (MCPyV PCR-negativity in CM2B4-positive tumors) supported the clinical utility of this antibody in the distinction of MCPyV-related MCC [19, 20, 29]. However, the sensitivity of this antibody (CM2B4) was slightly lower, as it identified approximately 70% of PCR-positive cases [19, 20, 40]. In 2012, the newer MCPyV antibody clone Ab3 was designed to enhance sensitivity [27].

In their initial study, Rodig et al. evaluated two MCPyV antibody clones in 57 cases and emphasized that Ab3 exhibited significantly greater sensitivity [27]. With the new clone, they detected 9 additional cases that CM2B4 had missed. However, CM2B4 remains commonly used in studies and routine practice, while reports on Ab3 are limited [21, 29, 30, 41]. The largest series comparing two antibody clones reported 90% positivity with Ab3 and 70% with CM2B4 [21]. In our series, approximately 80% of cases were positive with MCPyV clone Ab3 and 70% with clone CM2B4. Ab3 detected 5 additional cases that CM2B4 had missed, consistent with previous findings. In studies conducted to date, no cases have been reported that stained with CM2B4 but not with Ab3, as observed in our study.

In terms of specificity, studies indicate that both clones can occasionally exhibit immunoreactivity in non-neoplastic components [21, 36, 40]. With Ab3, a small subset of non-MCC skin cancers demonstrated focal and weak staining [21, 30]. In our study, although we did not test the antibodies on non-MCC tumors, we did not observe any aberrant staining in tumor surrounding tissues with either clone.

The extent and intensity of staining differ between the two clones. Ab3 has been reported to show higher extent and intensity, facilitating evaluation, while partial or weak staining has been reported in up to 25% of CM2B4-positive cases in literature [19, 26, 27, 41]. In our series, approximately 30% of CM2B4-positive cases showed low percentages or weak staining, complicating interpretation. Additionally, two of three cases with partial or weak Ab3 staining were negative with CM2B4. Similar findings of focal and weak staining with Ab3, yet negativity with CM2B4, have also been reported [30]. One study even described certain CM2B4 staining results as “uninterpretable” [20]. Based on these findings and our experience, we consider Ab3 to be easier to evaluate and more reliable than CM2B4, with higher sensitivity supported by current evidence.

Another finding that may vary geographically and etiologically in MCC is the expression status of CK20. CK20 plays a fundamental role in the diagnosis of MCC, and its perinuclear dot-like staining pattern is a distinctive and diagnostic feature for MCC [17]. However, it has been reported that approximately 10% of cases, particularly those negative for MCPyV, are CK20 negative [17, 18, 20, 22, 23, 42]. In our study, there were only two CK20 negative cases, which were also MCPyV negative. A study using next generation sequencing reported that CK20 and MCPyV-negative cases exhibit an ultraviolet (UV)-signature mutational profile [42]. Similarly, combined neuroendocrine carcinomas, which are mostly associated with squamous cell carcinoma, are MCPyV-negative and show a UV-signature mutational profile [1, 19]. Likewise, one of our MCPyV negative cases had Bowen disease overlying MCC indicating a UV radiation exposure.

Considering the key role of CK20 in MCC diagnosis, we investigated whether CK20 negativity might be influenced by factors beyond etiology. For this, we compared the commonly used Ks20.8 clone with the SP33 clone. To date, no studies have compared CK20 clones in MCC. In our series, two cases were negative with both clones, and no significant difference was observed in detecting positive cases. Diffuse staining was observed in a similar proportion of cases with both clones, although Ks20.8 showed stronger staining in about 40% of cases.

It is known that the typical DL staining pattern of CK20 in MCC can be accompanied by CS and M staining [19, 43]. In one-third of our cases, staining patterns differed between clones. Among these, Ks20.8 revealed additional CS staining in 40% and M staining in 60%. These variations highlight subtle enhancements between the clones. DL staining can be challenging to interpret, especially when weak or focal. In our series, we identified two cases where the DL staining was missed and incorrectly reported during routine practice (data not shown; author’s observation). This underscores the importance of selecting clones that produce sharper, more reliable staining to ensure accurate diagnosis.

We also noticed that SP33 stained necrotic areas in addition to viable tumor cells in 15 of our cases. In the same areas, no aberrant staining was observed with Ks20.8. Necrosis, observed in 70% of cases in our study, is a common feature of MCC due to its high grade. This aberrant SP33 staining could complicate evaluations, especially in resection specimens after neoadjuvant immunotherapy, where CK20 may play a role [44]. These findings highlight the need for careful consideration of clone selection.

This study has several limitations. The antibodies were only tested on skin and lymph nodes, and not on other tissues or non-MCC tumors. We also did not compare MCPyV antibody performance with PCR, which could have provided insights into their diagnostic accuracy. Lastly, our sample size may not fully represent the variability seen in routine practice. Future studies with larger cohorts and clone comparisons are needed to validate and expand these findings.

In conclusion, this study highlights the importance of selecting appropriate clones of MCPyV and CK20 antibodies in diagnosing MCC to enhance diagnostic accuracy. MCPyV antibody clone Ab3 demonstrated superior sensitivity and ease of interpretation compared to CM2B4, confirming its value in routine practice. While both CK20 clones Ks20.8 and SP33 showed comparable diagnostic performance in detecting positive cases, Ks20.8 exhibited stronger and more consistent staining patterns, which may aid in distinguishing challenging cases. The aberrant staining observed with SP33 in necrotic areas raises concerns, particularly in specimens from patients undergoing neoadjuvant therapy. These findings emphasize the need for further comparative studies to establish consensus on the optimal antibody clones for routine use in MCC diagnosis.

## Data Availability

No datasets were generated or analysed during the current study.
